# The Treatment of Pleurisy and Empyema

**Published:** 1893-07-29

**Authors:** 


### PADDINGTON GREEN CHILDREN'S HOSPITAL.

### The Treatment of Pleurisy and Empyema.

I. *Dry Pleurisy.*—This is treated by a few days rest in bed. The patient is put on a milk diet, including bread and butter, puddings, and eggs, and a simple saline is ordered, such as the following: ℞ Potas. citratis grs. v., liq. ammon. acetat. ʒi., syr. limonis, ♏x., aquam. ad. ʒij., sig. Two teaspoonfuls thrice daily. The affected part of the chest is painted with the liniment of iodine, or a hot fomentation containing two drachms of turpentine is applied for half an hour. The whole chest is then surrounded with a layer of cotton wool, which is fixed by means of a firm flannel bandage. If the pleuritic pain is very severe, two, three, or five drops of liquor morphiæ, according to the age of the child, are given, and may be repeated in two hours if necessary.

II. *Pleurisy with Effusion.*—When effusion is present, the treatment is directed to procuring its absorption as rapidly as possible. For this purpose the patient is kept absolutely at rest in bed. If there is much fever present, for example a temperature of 102 deg. to 103 deg., the diet is entirely fluid, and consists of milk, beef tea, and meat juice; if there is not much fever, milk puddings, eggs, and fish are allowed. The child is not stinted as regards the taking of liquids, such as plain water or barley water. A simple saline, as given above, is usually ordered, and to this five grains of iodide of potash are often added, with the view of promoting absorption of the effusion.

The local treatment consists in the repeated application of iodine to the affected part of the chest. The tincture is used in the case of infants, and for older children a mixture of equal parts of the tincture and the liniment is employed. This is painted on to the affected side, back and front, every morning until the skin becomes tender. After the application, the chest is wrapped in cotton wool and bandaged.

In most cases the above treatment will be sufficient, but in others the delayed absorption or increased exudation of fluid will lead to the consideration of the question of aspiration. No absolute rules can be laid down on this subject, and every case must be treated according to the special conditions present. Amongst the accepted indications for aspiration we may mention the following: (1) Continued fever, and no diminution of the effusion after ten days treatment as above; (2) the presence of a large amount of effusion, the dulness reaching, for example, as high as the second rib; (3) marked dyspnœa; (4) cardiac displacement, with a failing pulse; (5) the occurrence of bronchitis or pneumonia in the opposite lung.

The apparatus used in the withdrawal of the fluid is Potain's aspirator, which is now so well known that no description of it need be given. The needles and tubing are soaked for a couple of hours in carbolic acid lotion (one in twenty), and the bottle is washed out with the same fluid. Immediately before use the needles and tubing are syringed with boracic acid lotion, as the action of carbolic lotion is to produce coagulation of the albumin present in pleuritic exudation, and blocking of the tube would follow. The chest wall is thoroughly cleansed with soap and water, and carbolic lotion. The special site of puncture is in the seventh or eighth intercostal space, outside the line of the inferior angle of the scapula, when the effusion is extensive in amount; but if it is moderate, the needle is inserted at any part where the presence of fluid is manifested by absolute dulness on percussion. Two drachms of brandy are sometimes given before operation, so as to prevent any tendency to cardiac syncope. No anæsthetic is employed. The patient rests on the sound side at the edge of the bed, and is on no account allowed to sit up. The arm on the affected side is drawn well up over the head, so as to separate the ribs as far as possible. The receiving bottle having been partially exhausted, the operator makes the skin tense, and rests his left thumb on the upper border of the rib immediately below the space to be entered. A medium sized needle is then pushed sharply into the pleural cavity, with the point directed slightly upwards, and, guided by the thumb nail, passing in immediately above the rib. When it has been ascertained by the free movement in all directions that the end of the needle is in the pleural cavity, a moistened pad of salicylic wool is placed round the junction of needle and skin, so as to prevent the entrance of any air, and the trocar having been withdrawn, the stop cock is partially opened. It is very important that the pleural cavity should be emptied slowly, for a rapid evacuation leads to hæmorrhage from the pleura, and also to violent coughing, from the sudden expansion of the collapsed lung. The withdrawal of the fluid is regulated (1) by partial exhaustion of the receiving bottle, which can be repeated as often as necessary, and (2) by opening the stop cock so far as to allow a small stream of fluid to pass out, and not a violent rush. If a block takes place at the end of the needle, it can be cleared by means of the probe without allowing of the entrance of any air. Aspiration is usually kept up until the fluid ceases to flow, but if there is any tendency to syncope or dyspnœa, or if persistent coughing comes on, it is at once stopped. The needle is withdrawn, the puncture is closed with a layer of salicylic wool and collodion, over which a strip of plaster is placed, and the chest is again surrounded with cotton wool and a flannel bandage.

No attempt is made to empty the pleural cavity completely, and even when only a moderate amount of fluid has been removed, say ten to fifteen ounces, it is usually found that absorption of the remainder sets in after aspiration. The process is helped by continued painting of the affected side with iodine, and the internal administration of iodides. At this stage the best drug is the iodide of iron, in three or four grain doses, for anæmia is usually present in children who have had effusion into the pleural cavity. If the fluid re-accumulates, the operation of aspiration is repeated.

The patient is kept in bed for some time after aspiration, until it is clear from the increased movement on the affected side that the lung has re-expanded, and that the fluid is either absorbed or small in amount. Full nourishing diet, with cream, codliver oil, &c., is given, and the patient is made to expand the lungs as fully as possible by regulated gymnastic exercises. The cure is completed by a visit to the country or seaside for a few weeks.

(*To be continued.*)

